# Constructing a Computer Model of the Human Eye Based on Tissue Slice Images

**DOI:** 10.1155/2010/921469

**Published:** 2010-05-23

**Authors:** Peishan Dai, Boliang Wang, Chunbo Bao, Ying Ju

**Affiliations:** ^1^Institute of Biomedical Engineering, School of Info-Physics & Geomatics Engineering, Central South University, Changsha 410083, China; ^2^Department of Computer, Xiamen University, Xiamen 361005, China; ^3^Department of Computer, Fujian University of Technology, Fuzhou 350108, China

## Abstract

Computer simulation of the biomechanical and biological heat transfer in ophthalmology greatly relies on having a reliable computer model of the human eye. This paper proposes a novel method on the construction of a geometric model of the human eye based on tissue slice images. Slice images were obtained from an in vitro Chinese human eye through an embryo specimen processing methods. A level set algorithm was used to extract contour points of eye tissues while a principle component analysis was used to detect the central axis of the image. The two-dimensional contour was rotated around the central axis to obtain a three-dimensional model of the human eye. Refined geometric models of the cornea, sclera, iris, lens, vitreous, and other eye tissues were then constructed with their position and ratio relationships kept intact. A preliminary study of eye tissue deformation in eye virtual surgery was simulated by a mass-spring model based on the computer models developed.

## 1. Introduction

A computer model showing the fine anatomical structure of the human eye is the basis of the finite element analysis (FEA) of the eye and of computer simulation of eye surgery. In the recent years, many researchers have constructed human eye models to investigate projects in ophthalmology.

FEA using a human eye model before refractive corneal surgery is a very convenient way to quantitatively assess the effect of each surgical parameter on the optical outcome. Alastrué et al. [1] proposed a mechanical model of the human cornea and implemented this in a finite element context to simulate the effects of some typical surgical procedures, such as photorefractive keratectomy (PRK) and limbal relaxing incisions (LRI). Ooi et al. [2] constructed a three-dimensional (3D) radially symmetric boundary element model of the human eye in order to simulating changes in corneal temperature during treatment by laser thermokeratoplasty. In the past 100 years, the heat transfer phenomenon in the human eye has always been a major interest among researchers. A number of techniques have been used to measure temperature profile over the complete eye structure [3]. Ng and Ooi [4–6] focused on the heat transfer in the human eye and used human eye computer models to simulate thermal steady-state conditions based on properties and parameters reported in the open literature [5]. 

Ocular blunt trauma is a common ophthalmopathy disease. Uchio et al. [7] constructed a human eye model that has a fixated posterior chamber intraocular lens to determine the physical and mechanical conditions of an impacting air bag that can rupture an eye with such a lens. To determine conditions that can cause corneal rupture in a postradial keratotomy (RK) eye, and subsequently, to predict orbital deformation in subjects following a blunt injury, Uchio et al. [8] constructed a human eye model that has a postradial keratotomy. Al-Sukhun et al. [9] constructed an FEM of one of the orbits containing the globe based on CT-scan images. These research works have concluded that “finite element modeling is a powerful and invaluable tool to study the multifaceted phenomenon of orbital deformation.” 

Glaucoma is the second most common disease that causes blindness. It occurs when the normal fluid pressure inside the eyes slowly rises. Glaucoma is linked to intraocular pressure (IOP), so FEM is important in glaucoma research. Pan et al. [10] used FEM method to analyze the mechanical and fluidic performance of the valve in a glaucoma aqueous humor drainage device. Sigal and Ethier [11] attampted to describe how experiments and FEM modeling can be used to quantify the biomechanical environment within the lamina cribrosa (LC), and how this environment depends on IOP. Human eye models can also be used to analyze the eye accommodation mechanism [12–14] and other aspects of ophthalmology.

The present research is primarily based on the construction of geometric human eye models. However, the volume of the human eye is very small, the structure is very complex and the shape parameters are hard to measure. For these reasons, a three-dimensional computer modeling of the human eye is very difficult to accomplish. Three main modeling approaches are used for the present study. The first one is constructing eye models by a solid modeling method, wherein eye models are built using regular objects. The shortcoming of this method is that the model does not adequately simulate the complex shape of a real human eye. The second method is establishment of a parametric eye model using precise parameters obtained from medical experiments. However, it is difficult to precisely measure eye shapes since extensive knowledge on this topic remains very limited. The third method is building eye models using clinical image sequences, such as computer tomography (CT), magnetic resonance imaging (MRI), or ultrasound images. However, the current processing of in vivo medical imaging could not produce the required precision for human eye model construction. Most of the currently available geometric models of the human eye used in mechanical analysis have been built through solid modeling method, but there is a wide gap between the models and the actual geometric shape of the eye. 

We then propose the model construction approach based on tissue slice images as a means of building accurate 3D models of the human eye on a computer. 

In vitro eye slice images were used to construct 3D human eye models. The normal anatomical structure of the main eye tissues, such as the cornea, sclera, iris, lens, vitreous body, and optic nerve bunch, can be regarded as a rotator. The central section of the in vitro eye slice images was used to extract the contours of the main tissues of the eyeball. Feature points of the contours were then used to establish parameter models of the central section tissues of the in vitro eye. Geometric models of the main eye tissues was obtained by revolving the parameter models of the central section tissues. The sizes of the 3D models were adjusted to fit the actual size of real human eye tissues. If necessary, solid modeling methods were used to construct asymmetric eye tissues or tissues that could not be clearly identified from the slice images. Precise human eye models were then constructed by combining the models constructed through slice images and models created through solid modeling.

## 2. Methods

### 2.1. Data Acquisition

At present, most of the CT images, MIR images and the eye tissue images obtained from Virtual Human Project (VHP) [15–17] could not meet the precision required for constructing computer models of the human eye.

**Figure 1:**
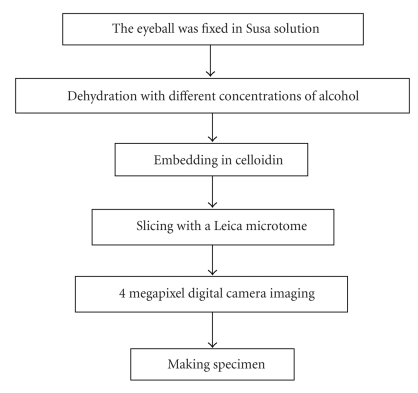
Human eye slice image production process.

**Figure 2:**
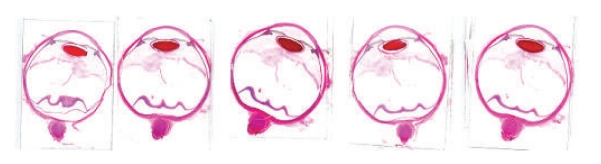
Human eye slice image sequence.

**Figure 3:**
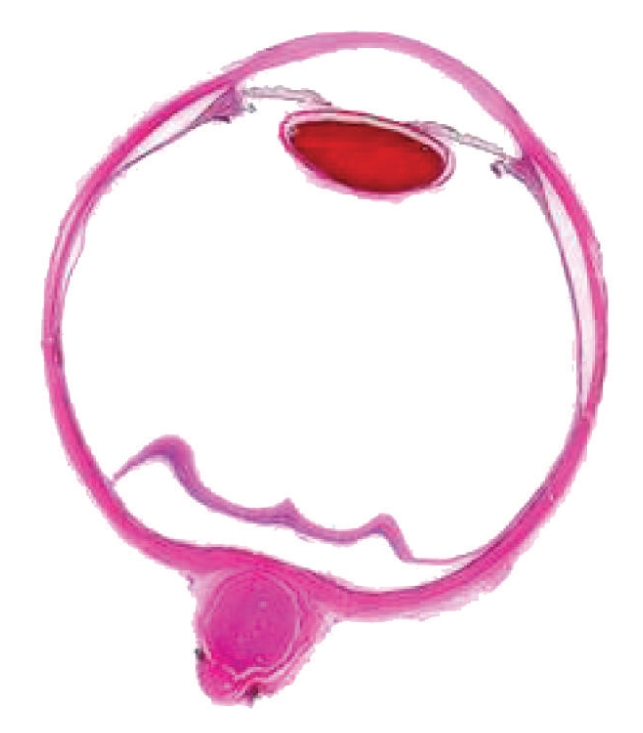
The image used to extract section features of the human eye.

Our research group obtained 10 in vitro Chinese eyeballs with the assistance of Southern Medical University and Shanghai University of Chinese Traditional Medicine and separately prepared fine slice images on two Chinese eyeballs in 2003 and 2004. Embryo specimen processing methods were used to prepare the eye slice images. The steps in the process are shown in Figure 1. The in vitro Chinese eyeballs were sliced with a Leica microtome, and each slice was photographed with a 4-megapixel digital camera. Two hundred forty eight (248) slices of the eyeball were made in 2003 and 646 slices in 2004. Some of the obtained image sequences are shown in Figure 2. Most of the slices showed precise eye structure, while some slices were seriously distorted. We used the central section of the in vitro eye slice image to extract the contours of the human eye. The image used to extract the features is shown in Figure 3.

### 2.2. Extracting the Contours and Feature Points of Eye Slice Images

The first step in constructing the human eye models is to extract the contours of the main eye tissues. It is the basis of the following steps, such as extracting feature points and symmetry axis of the contours.

#### 2.2.1. Slice Image Contour Extraction

**Figure 4:**
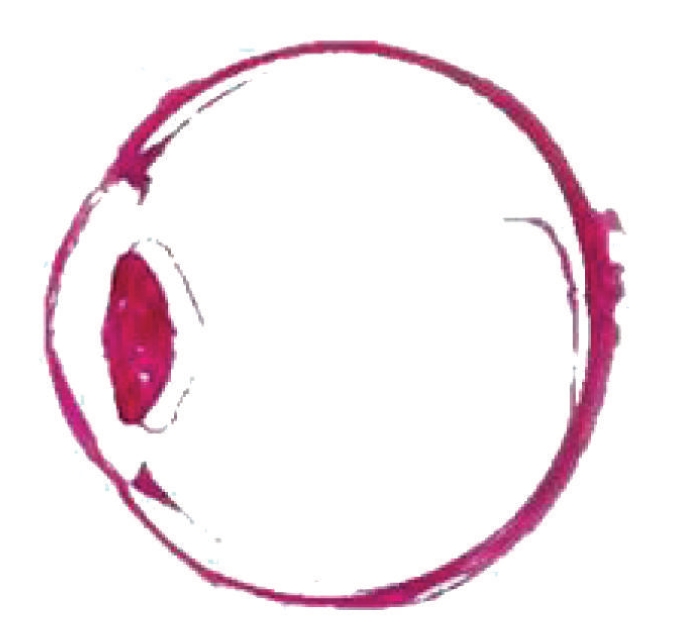
The segmentation results from region-growing algorithm.

To extract the contour of eyeball tissues, we tried popular medical image segmentation methods, but none of the results met our requirements for the construction of a computer model. Figure 4 shows one of the results from the region-growing algorithm. As indicated in the illustration, information on the iris contour and other important tissues were lost in the process. 

Therefore, it is necessary to use a more accurate method to extract the contours of important tissues. As the tissues in the slice images are surrounded by a light colored background, a level set algorithm may be a suitable method. The level set function of the traditional level set methods may generate oscillations and cups, adding to the uncertainty of further evolution. A common approach to avoid this gap is to repeat the step on initializing the level set function, Φ [18]. Reinitializing level set function Φ can be used to obtain a steady evolution curve, thus ensuring expected results. However, in practical operation, the step of reinitializing the level set may prove to be complex and time-consuming. Li et al. [19] presented a level set evolution algorithm that had no reinitialization step. The method allows a relatively large iteration step while speeding up curve evolution. The distance function is also easier to construct and use compared with the traditional distance function. 

We used the level set method proposed by Chunming et al. to segment the eye tissue contours. The evolution equation of the level set segmentation algorithm,

(1) $$E\left(\Phi \right)=\mu P\left(\Phi \right)+E_{g,\lambda ,\nu }\left(\Phi \right),$$

describes total energy, where P(Φ) is a penalty term (the approximate degree of level set function Φ and signed distance function) representing the internal energy,

(2) $$P\left(\Phi \right)=\int_{\Omega }\frac{1}{2}{\left(\left|\nabla \Phi \right|-1\right)}^{2}dx\;dy.$$

*E*_*g*,*λ*,*ν*_(Φ) represents the external energy, with the formula

(3) $$E_{g,\lambda ,\nu }\left(\Phi \right)=\lambda L_{g}\left(\Phi \right)+\nu A_{g}\left(\Phi \right),$$

where *λ* and *ν* are constants, *L*_*g*_(Φ) is the weighted length of level set, and *A*_*g*_(Φ) is weighted area. The formulas are as follows:

(4) $$\begin{array}{c} L_{g}\left(\Phi \right)=\int_{\Omega }g\delta \left(\Phi \right)\left|\nabla \Phi \right|dx\;dy,\\ A_{g}\left(\Phi \right)=\int_{\Omega }gH\left(-\Phi \right)dx\;dy. \end{array}$$

Taking the partial derivative of both sides of Formula (1), the following formula is obtained:

(5) $$\begin{array}{cc} \frac{\partial E}{\partial \Phi } & =-\mu \left[\Delta \Phi -\mathrm{div}\;\left(\frac{\nabla \Phi }{\left|\nabla \Phi \right|}\right)\right]\\ & \;-\lambda \delta \left(\Phi \right)\mathrm{div}\;\left(g\frac{\nabla \Phi }{\left|\nabla \Phi \right|}\right)-\nu g\delta \left(\Phi \right). \end{array}$$

Since

(6) $$\frac{\partial \Phi }{\partial t}=-\frac{\partial E}{\partial \Phi },$$

then

(7) $$\begin{array}{cc} \frac{\partial \Phi }{\partial t} & =\mu \left[\Delta \Phi -\mathrm{div}\;\left(\frac{\nabla \Phi }{\left|\nabla \Phi \right|}\right)\right]\\ & \;+\lambda \delta \left(\Phi \right)\mathrm{div}\;\left(g\frac{\nabla \Phi }{\left|\nabla \Phi \right|}\right)+\nu g\delta \left(\Phi \right). \end{array}$$

Here, *δ*(*x*) is replaced by the following smoother function:

(8) $$\delta_{\varepsilon }=\{\begin{array}{cc} 0, & \left|x\right|>\varepsilon ,\\ \frac{1}{2\varepsilon }\left[1+\cos\;\;\left(\frac{\pi x}{\varepsilon }\right)\right], & \left|x\right|\leq \varepsilon . \end{array}$$

Discrete Formula (7) can obtain the iterative formula of curve evolution

(9) $$\begin{array}{cc} \Phi_{i,j}^{k+1} & =\Phi_{i,j}^{k}+\tau \{\mu \left[\Delta \Phi -\mathrm{div}\;\left(\frac{\nabla \Phi }{\left|\nabla \Phi \right|}\right)\right]\\ & \;\;\;\;\;+\lambda \delta \left(\Phi \right)\mathrm{div}\;\left(g\frac{\nabla \Phi }{\left|\nabla \Phi \right|}\right)+\nu g\delta \left(\Phi \right)\}, \end{array}$$

where *k* is the iteration time, *τ* is the length of iteration step, and div(·) denotes divergence operation.

**Figure 5:**
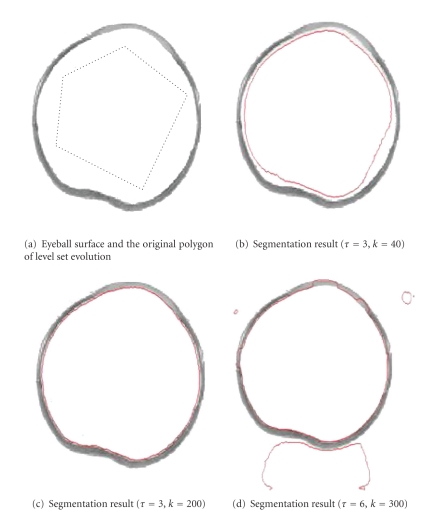
Extracting the inner surface of an eyeball through a level set algorithm.

We extracted the contours of the different eye tissues by level set segmentation methods. Figure 5 shows the segmentation results of the inner eyeball surfaces. Figure 5(b) shows the segmentation result with the iteration step length *τ* = 3 and iteration times *k* = 40. Figure 5(c) shows the segmentation result with parameters *τ* = 3 and *k* = 200 while Figure 5(d) shows the segmentation result with parameters *τ* = 6 and *k* = 300. The segmentation result in Figure 5(d) shows the portion that is more closely attached to the inner wall of eyeball. Figures 5(b) and 5(c) show the results with insufficiently and excessively curved evolution.

#### 2.2.2. Feature Point Extraction

The contours obtained from slice images could not be directly used to simulate the biomechanical and biological heat transfer in ophthalmology. First, the data acquisition process may lead to local deformation of the eye tissues. Second, the contours obtained from slice images could have a great deal of redundant data. If contour data were directly used to create geometric models of the human eye, the computational complexity would be very high. Third, even if there was no deformation in the contour, some local shapes may not tie in with the average shape of the eye tissues because of individual differences. Therefore, the extracted contour should be finely adjusted. 

The feature points that need to be extracted include (1) feature points of the rotation axis (2) key feature points of the contour shape.

**( 1) Rotation Axis Determination**

Geometric models of the main eye tissues were created by revolving the parameter models of the central section tissues around the rotation axis. Therefore, determining the rotation axis is very important in model construction. Each tissue may have asymmetric parts, although most tissues of the eyeball can be seen as a rotating body. The contours of the sclera, cornea, lens, iris, and so forth were all incorporated in the determination of the rotation axis in order to compensate for the impact of local asymmetry.

First, the Principle Component Analysis algorithm [20] was used to detect the symmetry axis. The principle of this algorithm is to turn a symmetry detection problem into a problem of the eigenvalue of a decomposed matrix. This requires the calculation of the covariance of the matrix composed of contour points set and the eigenvalue of the decomposed matrix in order to detect the symmetry.

The symmetry axis detected through the Principle Component Analysis algorithm may have some deviation with the rotation axis. The position of the symmetry axis was finely adjusted because it must pass through the centroid of the contour points. 

**Figure 6:**
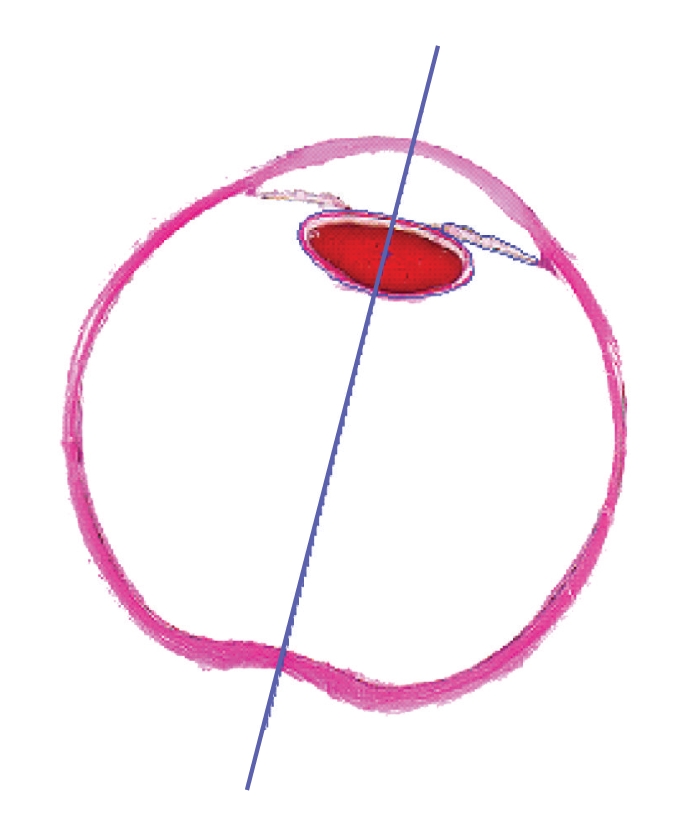
Identifying the symmetry axis of the human eye slice image.

The coordinates of the centroid point (*x*_*c*_,*y*_*c*_) are as follows:

(10) $$\begin{array}{c} x_{c}=\frac{1}{N_{\text{contour}}}\overset{N_{b}}{\underset{i=1}{\sum }}x_{i},\\ y_{c}=\frac{1}{N_{\text{contour}}}\overset{N_{b}}{\underset{i=1}{\sum }}y_{i}, \end{array}$$

where *N*_contour_ is the total number of the points on the contours of the eye tissues, and (*x_i_*,*y_i_*) are the coordinates of any contour point. Figure 6 shows the result of the symmetry axis detection; the blue line represents the symmetry detected using this method.

**( 2) Interactive Feature Point Extraction**

The feature points should present the features of the contours. Researchers have proposed many feature point extracting algorithms, which can be divided into two groups: polygonal approximation methods [21] and corner detection methods [22]. However, these methods have drawbacks.

Using these methods, the calculation of curvature and choice of the iteration step length and other parameters need a large number of experiments in order to obtain good feature points. The methods are also susceptible to noise. Meanwhile, the local shape deformation of contours will inevitably occur and such deformation could be easily identified by eye. Unfortunately, none of the present feature point extracting algorithms can eliminate this kind of deformation.

**Figure 7:**
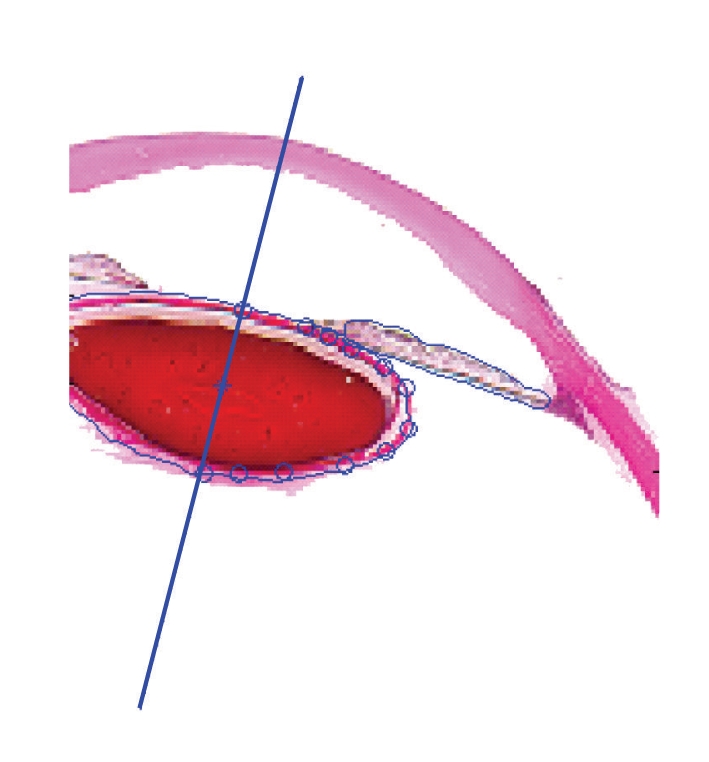
Interactive selection of the feature points.

Considering the specificity of the slice images, a human-computer interaction method was adopted to select the feature points of the contours. A software was designed to interactively select the feature points using a mouse. The coordinates of the marked feature points could then be obtained. The software has the capability of moving a feature point to a more precise position in the zoomed-in image. A distance judging method was used to estimate if the feature point could be seen as one feature point of the contour. Within an allowable range, the guiding principle is that the contour point nearest to the point selected with the mouse could be considered as a feature point. The points that were outside the range were considered as the feature points to make up the local deformation of the contours. The interactive selection of the feature point results is shown in Figure 7. The blue circles are the feature points selected to present the shape of lens.

**( 3) Determination of the Contour Points on the Symmetry Axis**

Note that the human eye models were created by rotating the section contours of the slice images around the symmetry axis. If the feature points of the contours do not include the contour points on the symmetry axis, there would be cracks around the symmetry axis in the 3D models. It is very difficult to select feature points exactly on the symmetry axis, although we can interactively select feature points much closer to the symmetry axis. However, since some feature points are much closer to the symmetry axis, the projection of these points on the symmetry axis could be seen as the feature points of the contours on the symmetry axis.

If a contour point (*x*_*k*_, *y*_*k*_) was on the symmetry axis, and the coordinates of the centroid point were (*x*_*c*_, *y*_*c*_), then the formula in deriving the symmetry axis would is

(11) $$\frac{x-x_{k}}{x_{c}-x_{k}}=\frac{y-y_{k}}{y_{c}-y_{k}}.$$

If the coordinates of an interactively selected point close to the symmetry axis were (*x_0_*, *y_0_*), then the perpendicular foot is the feature point on the symmetry axis. With the formula on distance between the beeline and the dot, we can obtain the coordinates of the feature point on the symmetry axis:

(12) $$\begin{array}{c} x_{0}^{\prime }=-\frac{b-b^{\prime }}{k-k^{\prime }}\\ y_{0}^{\prime }=kx+b, \end{array}$$

where

(13) $$\begin{array}{c} k=\frac{y_{c}-y_{k}}{x_{c}-x_{k}},\;\;k^{\prime }=\frac{-1}{k},\\ b=y_{k}-\frac{x_{k}}{x_{c}-x_{k}}\left(y_{c}-y_{k}\right),\;\;b^{\prime }=y_{0}-k^{\prime }x_{0}. \end{array}$$

In this way, for any point close to the symmetry axis, we can obtain the point that corresponds to the point on the symmetry axis. 

### 2.3. Spline Curve Fitting Using the Feature Points

After extracting the feature points, a cubic spline curve was used to connect the feature points to form the contours of one section.

Suppose that the number of the feature points is *n*, and the coordinates of these points were presented as (*x*_*i*_, *y*_*i*_) (*i* = 0,1, 2,…, *n*), then the cubic spline curve function is presented as *S*(*y*), and *M*_*i*_ (*i* = 0,1, 2,…, *n*) represents the second derivative at point *x*_*i*_ of *S*(*y*). Therefore, in the small interval [*y*_*i*_, *y*_*i*+1_], the interpolation function is

(14) $$\begin{array}{cc} S\left(y\right) & =M_{i}\frac{{\left(y_{i+1}-y\right)}^{3}}{6h_{i}}+M_{i+1}\frac{{\left(y-y_{i}\right)}^{3}}{6h_{i}}\\ & \;+\left(x_{i}-M_{i}\frac{h_{i}^{2}}{6}\right)\frac{y_{i+1}-y}{h_{i}}+\left(y_{i+1}-M_{i+1}\frac{h_{i}^{2}}{6}\right)\frac{y-y_{i}}{h_{i}}. \end{array}$$

The 3D geometric modeling of the main eye tissues was then created by revolving the section spline curves of the central section tissues.

### 2.4. Adjusting the Size of the Created Models

The created models incorporate spatial relationships, but the size needed to be adjusted to match the real size of an eyeball. The ratio of the slice image could be used as the foundation for the zoomed-in proportion. If the objective is to adjust the model size to match the real size of a human eye, the anteroposterior diameter and the vertical diameter of the human eye model could be adjusted according to the actual parameters of a human eye. The proportion of all of the characteristics of the models would then be matched with the actual size of the eyeball.

Apart from the method presented, the circumference of the eyeball can also be used as the basis for the zoom ratio. In the contours extracting step, we obtained the coordinates of the contour of the outer eyeball surface (*x*_*i*_, *y*_*i*_) (*i* = 1,…, *n*, where *n* denotes the number of the contour points). Using the Euler distance formula, we can then obtain the equatorial circumference of the eyeball slice image:

(15) $$d_{\text{perimeter}}=\overset{n-1}{\underset{i=1}{\sum }}\sqrt{{\left(x_{i+1}-x_{i}\right)}^{2}+{\left(y_{i+1}-y_{i}\right)}^{2}}.$$

The equatorial circumference of the slice image is 881.4 pixels. The normal value of the equatorial circumference reported for a Chinese human eye is about 74.5 mm [23]. Therefore, the zoom ratio of the eye model to a normal sized eye is

(16) $$R_{\text{eye}}=\frac{d_{\text{perimeter}}}{l_{\text{eye}}}=\frac{881.4\;\text{pixel}}{74.5\;\text{mm}}=11.8\;\left(\text{pixel}/\text{mm}\right).$$

### 2.5. Mesh Generation

The geometric models should be divided into small meshes in order to establish a physical model of the human eye in the computer. Numerous mesh-generating methods exist, such as the mesh mapping and node creation methods. At present, ANSYS is one of the commonly used FEM software programs. ANSYS can also provide for several mesh generation methods and can calculate differences in the generation. After the geometric model of eye tissues were imported into ANSYS, the material properties (i.e., Young Modulus and Poisson Ratio) could be set before or after mesh division. The base mesh can be chosen from an eight-note mesh (cubic), four-note mesh (tetrahedron), or other alternatives. The software includes a prewritten macro, ADAPT.MAC, which carries out auto adapted grid division.

### 2.6. Simulation of Eye Tissue Deformation

Based on the geometric models, a preliminary study of eye tissue deformation in virtual eye surgery was simulated by the mass-spring model. Herein, we utilized the deformation of the lens model as an example. The physical model used was simplified to a surface model because the biomechanical characteristics are very complex. The mass-spring model, which is simple and easy to use, was applied to simulate the biomechanical characteristics of the lens. To further simplify the simulation, the mesh model of the lens was also simplified by merging the vertices of the meshes into the surface model at 420 vertices and 762 triangles. 

Each vertex of the triangles corresponds to a particle. Meanwhile, the vertices of the triangles are connected by linear viscous springs. When one particle is affected by an external force, the force transmits to other particles through the springs connected with the particle. The change in particle displacement and the length of spring lead to the dynamic deformation of the spring mass model, simulating the deformation of the lens in virtual eye surgery.

The particle motion formula is obtained from Newton's second law of motion:

(17) $$m_{i}a_{i}=\overset{n}{\underset{j}{\sum }}g_{ij}-d_{i}\nu_{i}+f_{\text{exti}},$$

where *m*_*i*_ is the mass of the particle, *a*_*i*_, *ν*_*i*_ represent the acceleration and velocity of the particle, *d*_*i*_ represents viscosity coefficient, *f*_exti_ represents external force, and *g*_*ij*_ represents the stress between particle *i* and particle *j*. The particle motion trace can be obtained by solving the differential equation. The deformation of the lens in the external force state can then be simulated. 

## 3. Results

### 3.1. The Geometric Models of the Human Eye

**Figure 8:**
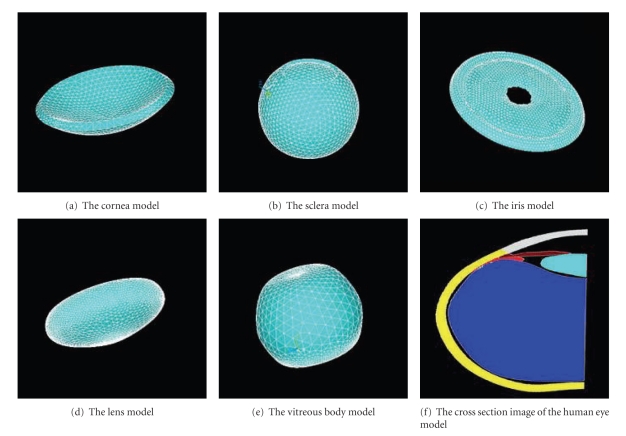
Human eye tissue models constructed through slice images.

Based on the methods presented, human eye geometric models were constructed. Figure 8 shows the geometric models of the cornea, sclera, iris, lens, and vitreous constructed in ANSYS. Figure 8(f) shows the sectional view of the human eye models. The gray part is the cornea; the yellow part is the sclera; the brown part is the iris; the red part is the ciliary body; the green part is the lens; the blue part is the vitreous. 

The FE elements of the constructed models are shown in Table 1.

### 3.2. Simulation of Eye Tissue Deformation

**Figure 9:**
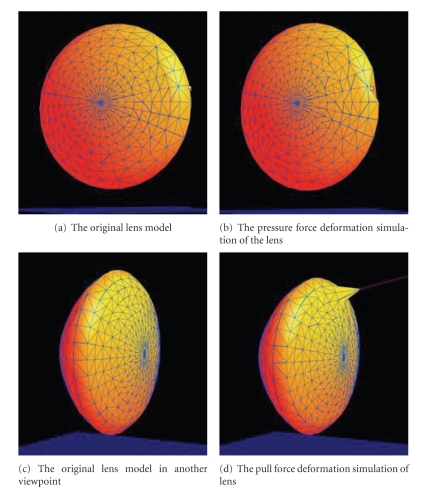
Simulation of human lens deformation.

The deformation of the physical model of the lens from the pressure and pull force was simulated, the results of which are shown in Figure 9. 

## 4. Discussion and Conclusion

**Figure 10:**
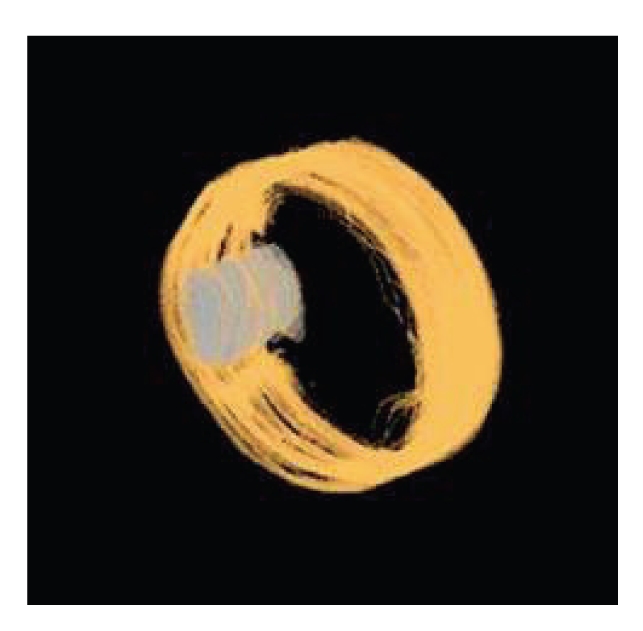
Reconstructed 3D model of the human eye by slice images using resampling.

The original purpose of our project was to reconstruct human eye models through in vitro eye slice images in order to obtain 3D reconstructions of the CT or MIR images using resampling and triangulation algorithms. However, when we obtained the eye slice images, we recognized that it was impossible to reconstruct eye models using these methods. Some slices were seriously distorted during the process of making the vitro eye slices because the volume of the eye is very small. Moreover, the registration of the slice images also presented much difficulty. Figure 10 shows the reconstructed 3D model of the human eye after image registration and volume rendering based on the obtained parts of the sliced images. Accordingly, we realized from the results that the usual 3D reconstruction algorithms may not work for the human eye slice images.

**Figure 11:**
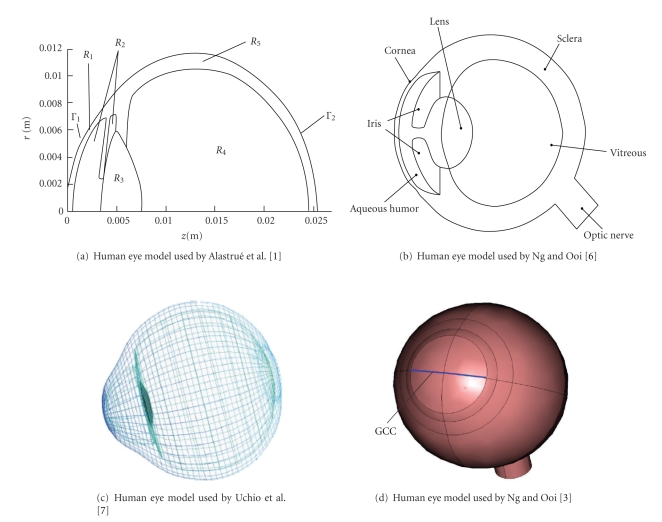
Human eye genomic models used in recent papers on computer simulation of biomechanical and biological heat transfer in ophthalmology.

Figure 11 shows several human eye geometric models created for FEM. As we can see, these models were all constructed by solid modeling methods. The geometric characteristics still present significant difference compared with the geometric characteristics of the real human eye. The difference of the models may affect the precision of the finite element analysis results.

In this paper, the geometric model of the cornea, sclera, iris, lens, vitreous, and other organizations were constructed, with their position relationships maintained. The 3D models constructed by the present study closely approximate the geometric characters of the real human eye. However, asymmetric eye tissues or tissues that cannot be clearly identified from the slice images continue to exist, and thus, solid modeling methods can be used to construct eye tissue models if needed. Based on the model constructed in this paper, many projects of biomechanical and biological heat transfer on ophthalmology can be analyzed using a computer. The model construction method can also be used to model a rotating body with a complex surface.

To test the models, we also constructed a physical model of the “lens-zonules-ciliary body” system. The system was used to simulate the accommodation mechanism of human crystalline lens by the finite element method. Simulation was conducted to study the Helmholtz's hypothesis [24] of the accommodation mechanism. The study showed that Helmholtz's hypothesis accords with current two facts of accommodation and basic rules during accommodation would not vary with the changing parameters of the model. Only the numerical range could be influenced by the parameters, indicating that the Helmholtz's hypothesis has been perfectly supported by the simulation. Thus, it has been partly proven that the analytical model presented in this paper can be used in the theoretical study of the human eye.

One drawback of the method is that the construction methodology was acquired from slice image, and as such, some shape information for each individual slice may have been lost in the process. 

In conclusion, we have proposed a methodology to construct computer models of the different components of the human eye. Refined geometric models of cornea, sclera, iris, lens, vitreous, and other eye tissues were constructed, with their position and ratio relationships kept intact. Eye tissue deformation in virtual surgery and the Helmholtz's hypothesis of the accommodation mechanism were simulated based on the computer models of the human eye. It has been partly proven that the analytical model presented in this paper can be used in the theoretical study of the human eye. The human eye model has a wide range of applications in the biomechanics or heat transfer simulation of the eye organ.

## Figures and Tables

**Table 1:** Grids (FE elements) of the constructed models.

Model name	Grids
The cornea model	2.842
The sclera model	3.576
The iris model	3.924
The lens model	4.123
The vitreous body model	3.762

## References

[1] Alastrué V, Calvo B, Peña E, Doblaré M (2006). Biomechanical modeling of refractive corneal surgery. *Journal of Biomechanical Engineering*.

[2] Ooi E-H, Ang W-T, Ng EYK (2008). A boundary element model of the human eye undergoing laser thermokeratoplasty. *Computers in Biology and Medicine*.

[3] Ng EYK, Ooi EH (2006). FEM simulation of the eye structure with bioheat analysis. *Computer Methods and Programs in Biomedicine*.

[4] Ooi EH, Ang WT, Ng EYK (2007). Bioheat transfer in the human eye: a boundary element approach. *Engineering Analysis with Boundary Elements*.

[5] Ng EYK, Ooi EH (2006). FEM simulation of the eye structure with bioheat analysis. *Computer Methods and Programs in Biomedicine*.

[6] Ng EYK, Ooi EH (2007). Ocular surface temperature: a 3D FEM prediction using bioheat equation. *Computers in Biology and Medicine*.

[7] Uchio E, Kadonosono K, Matsuoka Y, Goto S (2004). Simulation of air-bag impact on an eye with transsclerally fixated posterior chamber intraocular lens using finite element analysis. *Journal of Cataract and Refractive Surgery*.

[8] Uchio E, Kadonosono K, Matsuoka Y, Goto S (2004). Simulation of air-bag impact on an eye with transsclerally fixated posterior chamber intraocular lens using finite element analysis. *Journal of Cataract and Refractive Surgery*.

[9] Al-Sukhun J, Lindqvist C, Kontio R (2006). Modelling of orbital deformation using finite-element analysis. *Journal of the Royal Society Interface*.

[10] Pan T, Stay MS, Barocas VH, Brown JD, Ziaie B (2005). Modeling and characterization of a valved glaucoma drainage device with implications for enhanced therapeutic efficacy. *IEEE Transactions on Biomedical Engineering*.

[11] Sigal IA, Ethier CR (2009). Biomechanics of the optic nerve head. *Experimental Eye Research*.

[12] Liu Z, Wang B, Xu X, Wang C (2006). A study for accommodating the human crystalline lens by finite element simulation. *Computerized Medical Imaging and Graphics*.

[13] Abolmaali A, Schachar RA, Le T (2007). Sensitivity study of human crystalline lens accommodation. *Computer Methods and Programs in Biomedicine*.

[14] Martin H, Guthoff R, Terwee T, Schmitz K-P (2005). Comparison of the accommodation theories of Coleman and of Helmholtz by finite element simulations. *Vision Research*.

[15] Ackerman MJ (1998). The visible human project. *Proceedings of the IEEE*.

[16] Zhang S-X, Heng P-A, Liu Z-J (2006). Chinese visible human project. *Clinical Anatomy*.

[17] Park JS, Chung MS, Hwang SB, Shin B-S, Park HS (2006). Visible Korean human: its techniques and applications. *Clinical Anatomy*.

[18] Salac D, Lu W (2008). A local eemi-implicit level-set method for interface motion. *Journal of Scientific Computing*.

[19] Li C, Xu C, Gui C, Fox MD Level set evolution without re-initialization: a new variational formulation.

[20] Lu C, Zhang C, Wen F, Yan P (1999). Principle component analysis-based symmetry detection. *Acta Electronica Sinica*.

[21] Masood A (2008). Optimized polygonal approximation by dominant point deletion. *Pattern Recognition*.

[22] Gao X, Sattar F, Quddus A, Venkateswarlu R (2007). Multiscale contour corner detection based on local natural scale and wavelet transform. *Image and Vision Computing*.

[23] Jian G (2004). *Ophthalmology*.

[24] Helmholtz HV (1962). *Physiological Optics*.

